# Molecular characterization of infectious pancreatic necrosis virus strains isolated from the three types of salmonids farmed in Chile

**DOI:** 10.1186/s12985-017-0684-x

**Published:** 2017-01-31

**Authors:** René A. Manríquez, Tamara Vera, Melina V. Villalba, Alejandra Mancilla, Vikram N. Vakharia, Alejandro J. Yañez, Juan G. Cárcamo

**Affiliations:** 1 Instituto de Bioquímica y Microbiología, Facultad de Ciencias, Universidad Austral de Chile, Casilla 567, Valdivia, Chile; 2 Centro FONDAP, Interdisciplinary Center for Aquaculture Research (INCAR), Valdivia, Chile; 3 Institute of Marine and Environmental Technology, University of Maryland Baltimore County, Baltimore, 21202 USA

**Keywords:** IPNV, Birnavirus, Genotyping, Phylogenetic characterization, VP2, VP5

## Abstract

**Background:**

The infectious pancreatic necrosis virus (IPNV) causes significant economic losses in Chilean salmon farming. For effective sanitary management, the IPNV strains present in Chile need to be fully studied, characterized, and constantly updated at the molecular level.

**Methods:**

In this study, 36 Chilean IPNV isolates collected over 6 years (2006–2011) from *Salmo salar*, *Oncorhynchus mykiss*, and *Oncorhynchus kisutch* were genotypically characterized. Salmonid samples were obtained from freshwater, estuary, and seawater sources from central, southern, and the extreme-south of Chile (35° to 53°S).

**Results:**

Sequence analysis of the VP2 gene classified 10 IPNV isolates as genogroup 1 and 26 as genogroup 5. Analyses indicated a preferential, but not obligate, relationship between genogroup 5 isolates and *S. salar* infection. Fifteen genogroup 5 and nine genogroup 1 isolates presented VP2 gene residues associated with high virulence (i.e. Thr, Ala, and Thr at positions 217, 221, and 247, respectively). Four genogroup 5 isolates presented an oddly long VP5 deduced amino acid sequence (29.6 kDa). Analysis of the VP2 amino acid motifs associated with clinical and subclinical infections identified the clinical fingerprint in only genogroup 5 isolates; in contrast, the genogroup 1 isolates presented sequences predominantly associated with the subclinical fingerprint. Predictive analysis of VP5 showed an absence of transmembrane domains and plasma membrane tropism signals. WebLogo analysis of the VP5 BH domains revealed high identities with the marine birnavirus Y-6 and Japanese IPNV strain E1-S. Sequence analysis for putative 25 kDa proteins, coded by the ORF between VP2 and VP4, exhibited three putative nuclear localization sequences and signals of mitochondrial tropism in two isolates.

**Conclusions:**

This study provides important advances in updating the characterizations of IPNV strains present in Chile. The results from this study will help in identifying epidemiological links and generating specific biotechnological tools for controlling IPNV outbreaks in Chilean salmon farming.

## Background

Infectious pancreatic necrosis virus (IPNV) is the causative agent of a contagious fish disease by the same name and is responsible for economic losses in the aquaculture industry worldwide. In 2015, this viral agent represented about 30% of disease diagnoses reported from Chilean salmon farming centers and caused about 8% of pathogen-associated mortalities [1]. Infections with IPNV mainly occur in the freshwater or fry first-feeding stages, but fingerlings of most salmonids can also become infected [2]. The transfer of young salmon to saltwater is a particularly stressful stage, and high mortalities associated with IPNV outbreaks are commonly reported. Notably, infected fish that overcome IPN remain asymptomatic carriers, with some authors reporting that the virus continues replicating in hematopoietic tissues [3–5]. Carrier fish could possibly be the most important mechanism through which this virus is maintained and remains contagious.

**Fig. 1:**
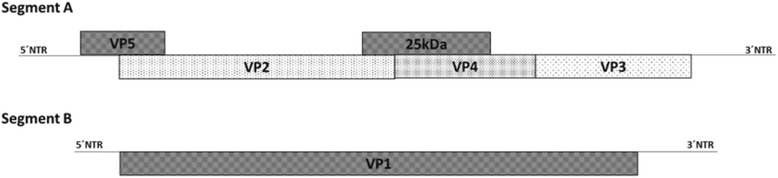
IPNV genomic map. Segment A gene structure showing the encoded proteins, the major open reading frame encoding the polyprotein (NH2-pVP2-VP4-VP3-COOH), a second overlapping open reading frame that encoded no structural VP5 protein, and the putative 25 kDa protein codifed from the third overlapping open reading frame described by Shivappa et al. [12]. Segment B encoded VP1, the RNA-dependent RNA polymerase

IPNV is the prototype virus of the *Birnaviridae* family; the virion consists of two genome segments of double-stranded RNA (termed A and B) that are encased in a non-enveloped icosahedral capsid [6]. Segment B of the IPNV genome is monocistronic, contains approximately 2783 nucleotides, and encodes for the RNA-dependent RNA polymerase VP1. In turn, segment A contains approximately 3097 nucleotides, is bicistronic and has an open reading frame (ORF) that encodes a 106-kDa polyprotein (NH2-pVP2-VP4-VP3-COOH) (Fig. 1). This polyprotein is co-translationally cleaved by viral protease 4 (termed VP4 or NS) [7] to generate the viral capsid proteins pVP2 (comprised by amino acids 1 to 512), VP3 (756 to 1012), and protease VP4 (513 to 755) [8, 9]. Subsequently, pVP2 is further processed at the C terminus to obtain the mature VP2 protein [10, 11]. In Sp IPNV serotypes, an ORF coding for a putative 25 kDa protein exists between the VP2 and VP4 coding regions that contains a nuclear localization signal (NLS) [12].

The VP2 gene encodes the principal capsid protein of IPNV, which is responsible for virulence and contains all neutralizing epitopes. The key residues involved in virulence are Thr, Ala, Thr/Ala, and Tyr/His at positions 217, 221, 247, and 500, respectively [12–14].

Also in relation to segment A, some IPNV isolates present a smaller ORF that partially overlaps and precedes the major ORF. This smaller ORF encodes a single peptide termed VP5 (Fig. 1), which ranges in size from 15 kDa to 3.3 kDa [15–17]. VP5 might also participate in the virulence mechanism, but studies with VP5-deficient mutants gave a similar mortality that VP5-producing strains [18]. Likewise, some IPNV isolates lack the reading-frame for VP5 [19, 20], while some highly virulent IPNV strains have truncated forms of this peptide [17]. While these results seem to exclude VP5 as a factor linked to virulence, discrepancies between studies remain. Therefore, additional research is required to fully elucidate the roles of VP2 and VP5 in the virulence of IPNV infection. The VP5 sequence does contain the Bcl-2 homology (BH) domains BH1, BH2, BH3, and BH4 typical of class I and II Bcl-2 family proteins [21, 22]. Accurate understandings on the role of these sequences in the cellular infection mechanism remain lacking, as is also the case for the functional effects of structural motif variations in VP5.

Serological studies have identified two aquatic birnavirus serogroups, A and B [23]. IPNV belongs to serogroup A, which contains nine serotypes (A1 to A9). However, perhaps the most significant contribution towards the classification of IPNV was proposed by Blake et al. [24] through phylogenetic analysis of deduced amino acid sequences. This analysis revealed six genogroups that generally correlate with the geographical origin of the virus and previous serological classifications. Subsequent phylogenetic analysis conducted by Nishizawa et al. [25] revealed the existence of a new genogroup,G.7, for Japanese *aquabirnavirus* isolates. Therefore, according to the classification proposed by Blake et al. [24], genogroup 1 is composed of USA isolates (A1) and Jasper strains from Canada; genogroup 2 is composed of isolates from Europe (A3) and Asia; genogroup 3 is composed of two isolates from Canada (C1) (A6) and the European Tellina strain (A5); genogroup 4 is composed of isolates Canada 2 (A7) and Canada 3 (A8) isolates; genogroup 5 is composed of five European isolates and one isolate from Asia (A2); genogroup 6 is composed by only the Hecht isolate (A4); and genogroup 7 is composed of isolates mainly from Japan and Korea [25, 26].

Several studies link Chilean IPNV isolates with sero- or genotypes from North America and Europe. Early reports found that Chilean IPNV isolates from rainbow trout were similar to the North American serotype VR-299, but this generated debate regarding the possible presence of serotype Sp, which is of European origin [27, 28]. According to the classification of Blake et al. [24], serotypes VR-299 and Sp belong to genogroups 1 and 5, respectively. Later, Mutoloki and Evensen [29] grouped some Chilean isolates from Atlantic salmon with Norwegian and Irish isolates, a classification consistent with the Sp serotype and genogroup 5. Likewise, researchers from Valparaiso, Chile [30, 31] phylogenetically analyzed five and 18 IPNV samples, respectively, from different locations from the south-Austral region of the country, and, as in previous studies, most of the sequenced isolates were of European origin (genogroup 5), whereas the remaining were of North American origin (genogroup 1). Furthermore, real-time RT-PCR assays based on Univeral ProbeLibrary probes successfully identifies and discriminates between Chilean isolates from both IPNV genogroups [32], and the present study more deeply assessed several of these same isolates.

Chile currently has approximately 241 freshwater-phase salmonid farms and 485 seawater salmonid farming concessions, resulting in 726 salmonid farming centers nationwide. Considering the wide prevalence of this industry, there is a persisting need to widely extend the number of analyzed IPNV isolates. Additionally, novel studies should be more extensive and include many more sampling points, a vast amount of different isolates, various salmonid sources, and diverse water types, in addition to ideally collecting samples over an extended period of time and over a wide territory. Studies should also try to combine analysis techniques to expand on the available knowledge regarding molecular underpinnings, genome characterization, VP5 characterization, VP2 virulence markers, sequence and motif conservations, and phylogeny. Such studies would be highly relevant in fully elucidating the roles of VP2 and VP5 in the virulence mechanisms associated with IPNV infection.

To advance the available knowledge on IPNV, this study carried out genotypic and phylogenic characterizations for 36 Chilean IPNV isolates collected over a 6 year period (2006–2011). Additional analyses of VP2 and comparative genotyping of VP5, including the putative 25 kDa protein, were also performed for all isolates.

## Methods

### Virus isolation and cell cultures

For growth and titration of IPNV from sampled tissues, the RTG2 cell line, from rainbow trout gonads (ATCC CCL-55) and CHSE-214, from Chinook salmon (*Oncorhynchus tshawytscha*) embryo cells (European Collection of Cell Cultures, ECACC 91041114) were used. Cells were propagated in Leibovitz’s L-15 culture medium (Life Technologies) supplemented with 10% fetal bovine serum. The incubation temperature used for growth was 17 °C. Confluent cell layers were sub-cultured twice a week.

The virus isolates were kindly provided by the company Biovac S.A., and the Institute of Pathology, Faculty of Veterinary Sciences, Universidad Austral de Chile. According to the information provided by them, these samples were obtained from *S. salar*, *O. mykiss*, and *O. kisutch*, in different locations along the southern Chilean, from 2006 to 2011 (Table 1). The virus isolates were inoculated on monolayers of RTG2 cell cultures. Once an extensive cell cytopathic effect occurred (3–7 days post-infection), the cell culture medium was harvested and stored in aliquots until use. Concomitantly, the viral cultures were titrated by an end-point dilution assay, using CHSE-214 cells.

**Table 1:** The Chilean infectious pancreatic necrosis virus isolates were obtained from different geographical regions and salmonid species. Additionally, the tissue, stage, weight, water source, and year of the isolate are reported

Isolate code	Tissue used for isolation	Geographical area^a^	Species	Stage	Water source	Year	Accession number
1096-BC1	Mix (kidney, liver, spleen)	Aysén R. (XI Region)	*O. kisutch*	Juvenile	NA	2009	KX523824
1096-BC2	Mix (kidney, liver, spleen)	Comau Fjord (X Region)	*S. salar*	Juvenile	Freshwater	2010	KX523802
1096-BC3	Mix (whole fish)	Maule R. (VII Region)	*S. salar*	Alevin	Freshwater	2009	KX523827
1096-BC4	Mix (kidney, liver, spleen)	Comau Fjord (X Region)	*S. salar*	Alevin	Freshwater	2010	KX523803
1096-BC5	Mix (kidney, liver, spleen)	Aysén R. (XI Region)	*O. kisutch*	Juvenile	NA	2009	KX523816
1096-BC6	Mix (kidney, liver, spleen)	Los Angeles (VIII Region)	*O. mykiss*	Alevin	Freshwater	2009	KX523804
1096-BC7	Mix (kidney, liver, spleen)	Comau Fjord (X Region)	*O. mykiss*	Juvenile	Freshwater	2010	KX523818
1096-BC8	Mix (kidney, liver, spleen)	Rupanco Lake (X region)	*O. mykiss*	Juvenile	Freshwater	2010	KX523828
1096-BC9	Mix (kidney, liver, spleen)	Reloncaví Cove (X Region)	*S. salar*	Adult	Freshwater	2010	KX523829
1096-BC10	Mix (kidney, liver, spleen)	Rupanco Lake (X region)	*O. mykiss*	Juvenile	Freshwater	2010	KX523801
1096-BC11	Mix (kidney, liver, spleen)	Cisnes (XI Region)	*S. salar*	NA	Seawater	2010	KX523819
1096-BC12	Mix (kidney, liver, spleen)	Calbuco (X Region)	*S. salar*	Alevine	Freshwater	2010	KX523800
1096-BC13	Mix (kidney, liver, spleen)	Reloncaví Estuary (X Region)	*S. salar*	Adult	Estuary	2010	KX523833
1096-BC14	Mix (kidney, liver, spleen)	Comau Fjord (X Region)	*S. salar*	Alevine	Freshwater	2010	KX523821
1096-BC15	Mix (kidney, liver, spleen)	Reloncaví Estuary (X Region)	*S. salar*	Adult	Estuary	2010	KX523830
1096-BC16	Mix (kidney, liver, spleen)	Cisnes (XI Region)	*S. salar*	Smolt	Seawater	2010	KX523805
1096-BC17	Mix (kidney, liver, spleen)	Nehuentue (VIII Region)	*S. salar*	Alevin	Freshwater	2011	KX523822
1096-BC18	Mix (kidney, liver, spleen)	Calbuco (X Region)	*S. salar*	Alevin	Freshwater	2010	KX523820
1096-V1	Mix (whole fish)	Araucanía R. (IX Region)	*O. mykiss*	Alevin	Freshwater	2006	KX523809
1096-V2	Mix (kidney/spleen)	Magallanes R. (XII Region)	*S. salar*	Pre-smolt	Freshwater	2009	KX523810
1096-V3	Mix (kidney/spleen)	Magallanes R. (XII Region)	*S. salar*	Pre-smolt	Freshwater	2009	KX523811
1096-V4	Mix (kidney/spleen)	Los Ríos R. (XIV Region)	*O. kisutch*	Pre-smolt	Estuary	2008	KX523799
1096-V5	Mix (kidney/spleen)	Los Ríos R. (XIV Region)	*S. salar*	Smolt	Estuary	2008	KX523807
1096-V6	Mix (kidney/spleen)	Los Lagos R. (X Region)	*O. mykiss*	Smolt	Freshwater	2006	KX523808
1096-V7	Mix (whole fish)	Los Lagos R. (X Region)	*O. mykiss*	Alevin	Freshwater	2006	KX523825
1096-V8	Mix (kidney/spleen)	Los Ríos R. (XIV Region)	*O. mykiss*	Pre-smolt	Estuary	2007	KX523806
1096-V9	Mix (kidney/spleen)	Los Lagos R. (X Region)	*S. salar*	Pre-smolt	Estuary	2006	KX523826
1096-V10	Mix (kidney/spleen)	Los Lagos R. (X Region)	*S. salar*	Alevin	Freshwater	2006	KX523823
1096-BC20	Mix (kidney, liver, spleen)	Aysén R. (XI Region)	*S. salar*	NA	NA	2009	KX523817
1096-BC21	Mix (kidney, liver, spleen)	Araucanía R. (IX Region)	*O. kisutch*	Alevin	Freshwater	2009	KX523813
1096-BC22	Mix (kidney, liver, spleen)	Aysén R. (XI Region)	*S. salar*	Adult	Seawater	2009	KX523812
1096-BC23	Mix (kidney, liver, spleen)	Aysén R. (XI Region)	*O. kisutch*	Juvenile	Freshwater	2009	KX523815
1096-BC24	Mix (kidney, liver, spleen)	Aysén R. (XI Region)	*O. kisutch*	Alevin	Freshwater	2009	KX523814
1096-BC25	Mix (kidney, liver, caeca)	Los Ríos R. (XIV Region)	*S. salar*	Alevin	Freshwater	2010	KX523832
1096-BC26	Mix (kidney, liver, caeca)	Araucanía R. (IX Region)	*O. kisutch*	Alevin	Freshwater	2009	KX523831
1096-BC27	NA	NA	NA	NA	NA	2010	KX523834

NA: Not available. ^a^Chile is geopolitically dived from north to south into 15 distinct regions equally recognized by Roman numerals or written names. The regions included in this study are as follows: VII = Maule Region; VIII = Biobío Region; IX = Araucanía Region; X = Los Lagos Region; XI = Aysén Region; XII = Magallanes Region; and XIV = Los Ríos Region

### Viral RNA extraction and reverse-transcription (RT)

Viral RNA was isolated with the E.Z.N.A. Total RNA Kit (Omega Bio-tek) according to the manufacturer’s instructions. For cDNA synthesis, 10 μl of the RNA suspension were subjected to reverse-transcription using the M-MLV Reverse Transcriptase Kit (Life Technologies) and random primers according to the manufacturer’s instructions. The cDNA was stored at −20 °C until use.

RT-qPCR used primers PrD1 5′-AAAGCCATAGCCGCCCATGAAC-3′ and PrD2 5′- TCTCATCAGCTGGCCCAGGTAC-3′ to detect IPNV-positive samples [33] using a StepOnePlus™ System (Thermo Fisher, USA). Reactions (10 μl) included 1 μl of template cDNA, 0.5 μM primers and components provided by Fast SYBR Green Master Mix (Thermo Fisher, USA). Cycle conditions were for 20 s at 95 °C, followed by 40 cycles including denaturation for 3 s at 95 °C and annealing for 10 s at 60 °C. Melting curve analysis of amplification products was performed at the end of PCR to confirm that only one PCR product was amplified and detected. For partial sequencing of the VP2 gene in the different isolates, PCR was applied using KOD Hot start DNA Polimerasa (CALBIOCHEM/USA) and the primers PrA1 5′-TGAGATCCATTATGCTT CCCGA-3′ and PrA2 5′-GACAGGATCATCTTGGCATAGT-3′. Cycle conditions were for 5 min at 95 °C, followed by 40 cycles including denaturation for 30 s at 95 °C, annealing for 30 s at 60 °C and final extension for 30 s at 72 °C as described by Blake et al. [33].

### Nucleotide sequencing and analysis of VP2

PCR products of 1180 base pairs were recovered from agarose gel, purified using the E.Z.N.A. Extraction Kit (Omega Biotek), and cloned into the pCR2.1 vector using a TOPO TA Cloning Kit for Sequencing (Invitrogen by Life Technologies). The pCR2.1 plasmid, with the 36 different inserts, were sent to Macrogen Inc. (Korea) for sequencing using the M13 universal primers (M13F: 5′-GTAAAACGACGGCCAGT-3′ and M13R: 5′-AACAGCTATGACCATG-3′). The obtained nucleotide sequences were assembled using DNA Baser v4.12, and the deduced amino acid sequences were obtained using the online ExPASy tool (http://web.expasy.org/translate/). The obtained VP2 sequences and Genbank strains representative of all seven IPNV genogroups and of the previously reported Chilean IPNV isolates [24, 29, 30] were included in comparative and phylogenetic analyses.

The deduced amino acid sequences were aligned using ClustalW in the Bioedit software and analyzed in relation to previously described amino acid virulence markers for IPNV [12–14]. Pairwise distances were calculated for the percentage of nucleotide and amino acid identities of total sequences. Phylogenetic and molecular evolutionary analyses were conducted using the MEGA v5 software [34]. Prior to the construction of the phylogenetic tree, the best model for DNA/Protein to VP2 sequences was computed. Then, phylogenetic analysis was performed using the deduced amino acid sequences, with evolutionary history inferred by using the maximum likelihood method based on the JTT matrix-based model [35]. One thousand bootstrap replicates were performed, and a condensed phylogenetic tree was constructed for the genogroups comparison with previously described reference strains (Table 2).

**Table 2:** Reference aquabirnavirus strains used

Strain	Geographical origin	Host	Genogroup	Serotype	GenBank accession N°
WB	Maine, USA	Trout	1	A1	AF342727
Sp	Denmark	Trout	5	A2	AF342728
Ab	Denmark	Trout	2	A3	AF342729
He	Germany	Pike	6	A4	AF342730
Te	England	Tellina	3	A5	AF342731
C1	Canada	Trout	3	A6	AF342732
C2	Canada	Trout	4	A7	AF342733
C3	Canada	Arctic char	4	A8	AF342734
Jasper	Canada	Trout	1	A9	AF342735
YTAV	Japan	Yellowtail	7		AY283781

### Nucleotide sequencing and analysis of VP5

The previously synthesized viral cDNA was additionally used to amplify the region encoding VP5. The primers used in PCR analysis (VP5F: 5′- TCCGTCGATGGCGAAAGCCC-3′ and VP5R: 5′- CTCCACCTCAGACAGACTGCC -3′) generated a product of 460 base pairs. This product was recovered from agarose gel, purified using the E.Z.N.A. Extraction Kit (Omega Biotek), and cloned into the pCR2.1 vector using a TOPO TA Cloning Kit for Sequencing (Invitrogen Life Technologies). The pCR2.1 vectors with the different inserts were sent to Macrogen Inc. (Korea) for sequencing, using a similar analysis to the one performed for VP2. To obtain the complete deduced amino acid sequences of VP5 isoforms longer than 269 amino acid residues (around 29.6 kDa), the nucleotide sequences obtained for VP5 were overlapped to the VP2 nucleotide sequences previously obtained. To assess if the VP5 sequences of the collected Chilean isolates contained transmembrane domains, public transmembrane domain predictors (TMpred and TOPCONS) were run with VP5 amino acid sequences. Furthermore, to determine the presence of a cationic cluster for plasmatic membrane tropism, the VP5 sequences were aligned and analyzed for the presence of polycationic regions using the ClustalW tool and the VP5 sequence of the infectious bursal disease virus [36].

#### WebLogo analyses

To study the variability of the deduced amino acid sequences of four BH domains in VP5, the VP5 sequences of Chilean IPNV isolates were aligned using the MUSCLE software [37]. The obtained results were run in the online WebLogo tool (http://weblogo.berkeley.edu/), where each residue of the BH domains is represented as a stack of one letter for each amino acid and where size indicates sequence conservation at each position. The sequences for each VP5 BH domain of Chilean IPNV isolates were compared with conserved BH1, BH2, BH3, and BH4 sequences described for these domains, which included the BH domains of IPNV E1-S (Ab strain), MABV Y-6, and other Bcl-2-related proteins sharing homology in one or more of these domains, in accordance with previously described methodologies [38, 39].

### Analysis of the ORF for putative 25 kDa protein

Nine of the 36 Chilean IPNV isolates (1096-BC1, BC2, BC4, BC6, BC10, BC12, BC16, and 1096-V4 and -V8) were subjected to full sequencing of segment A. This provided information on the possible presence of the ORF for the putative 25 kDa protein between the VP2 and VP4 coding regions. The nucleotide sequence of the putative 25 kDa protein described for isolate Sp103 (Accession number AY354519.1) was used in multiple alignment analyses with the online ClustalW tool. Then, the deduced amino acid sequence of each ORF was identified using the online ExPASy tool [40]. To determine possible NLS, the online CNLS Mapper (http://nls-mapper.iab.keio.ac.jp/cgi-bin/NLS_Mapper_form.cgi) and NLStradamus [41] tools were used. Moreover, to identify possible mitochondrial location signal sequences, the online predictor TargetP 1.1 was used (http://www.cbs.dtu.dk/services/TargetP/).

## Results

**Fig. 2:**
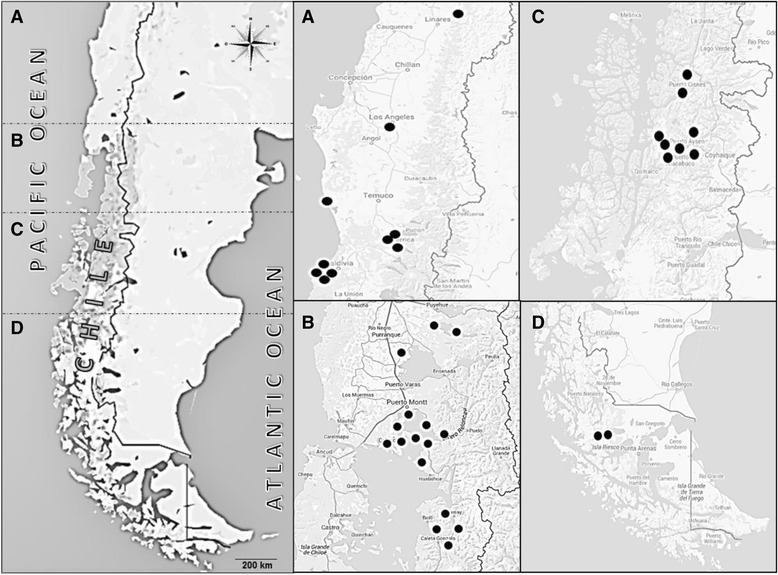
Map of the sampling sites in extreme-south of Chile. Vertical panel to the left depicts part of Chile divided in four sections. The panels to the right show greater detail of Chilean territory in sections before defined, pointing out the sampling sites (black circles)

A virus collection with 36 Chilean IPNV isolates was constituted. These isolates were sampled from the three most important farmed salmonids in Chile: Atlantic salmon (*S. salar*), rainbow trout (*O. mykiss*), and coho salmon (*O. kisutch*). These fish were sampled from waters with different salinities (freshwater, estuaries, and seawater) and over a wide expanse of territory extending along central to southern Chile (35° to 53° S; Fig. 2).

**Fig. 3:**
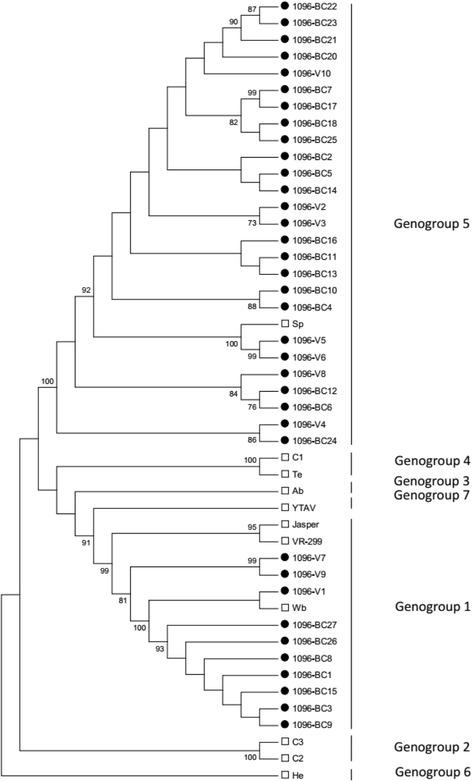
A condensed phylogenetic tree showing the relationships between Chilean IPNV isolates and reference strains of 9 IPNV serotypes plus the *Aquabirnavirus* YTAV, based on nucleotide sequences of VP2. The tree was constructed through the maximum likelihood method using 1000 bootstrap replications. Black circles correspond to the Chilean IPNV isolates, and white squares correspond to the reference strains included in the analysis

Using molecular phylogenetic analyses, 10 of the 36 Chilean IPNV isolates were classified within genogroup 1 (around 28% of all isolates; Fig. 3), a group that also includes the North American isolates Jasper and West Buxton (Table 2). The remaining 26 IPNV isolates were classified within genogroup 5 (72%), close to the European isolate Sp. The 10 Chilean isolates belonging to genogroup 1 had nucleotide identity percentages ranging from 76.9 to 99.4% (Table 3), whereas, those within genogroup 5 had nucleotide identities ranging from 87.7 to 100% (Table 4), with isolates BC-11 and BC-13 being identical. Nucleotide sequence comparisons between the VP2 of Chilean isolates and their respective reference strains found that the highest nucleotide variability between the 10 isolates of genogroup 1 and the West Buxton strain is 19.7% (Table 3). This variability was around 11.0% for the 26 isolates of genogroup 5 as compared to the Sp strain (Table 4). The percentages of identity for deduced amino acid sequences between the isolates of genogroup 1 were higher than nucleotide identities (90.61 to 99.45%; Table 3), and this situation was even more marked for isolates in genogroup 5, with several isolates showing 100% amino acid identity (Table 4).

**Table 3:**
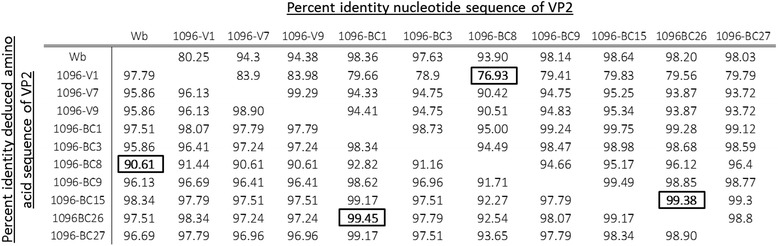
Pairwise comparison of percent identity between the nucleotide and deduced amino acid sequences of VP2 from IPNV isolates belonging to genogroup 1. The West Buxton strain (GenBank Accession Number AF342727) was included as a reference. The highest and lowest identity percentages are highlighted in boxes

**Table 4:**
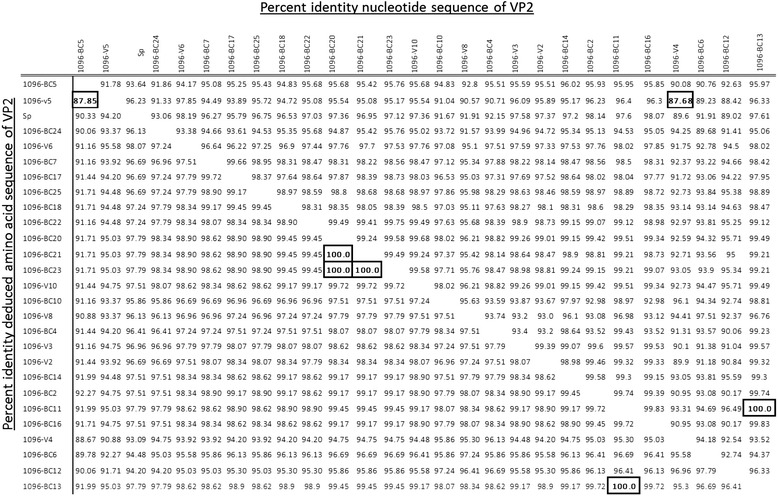
Pairwise comparison of percent identity between the nucleotide and deduced amino acid sequences of VP2 from IPNV isolates belonging to genogroup 5. The Sp strain was included as a reference (GenBank Accession Number AF342728). The highest and lowest identity percents are highlighted in boxes, as are isoltes with full identity (100%)

Despite only having precise mortality (%) information for two isolates, the VP2 amino acid residues with described virulence involvement in the Sp serotype, were compared between the deduced amino acid sequences of genogroup 5 isolates. This analysis revealed that 15 presented the Thr, Ala, and Thr residues at positions 217, 221, and 247, respectively (Table 5). Sequencing beyond amino acid position 500 was only accomplished in eight of these isolates, with seven presenting Tyr in this position and one presenting His. Similarly, comparative analysis of the 10 genogroup 1 isolates revealed nine to have Ala, Thr, and Ala at positions 217, 221, and 247, respectively. Only one isolate was sequenced beyond position 500, showing Arg in this position (Table 5).

**Table 5:** Amino acid variations in the VP2 region of the of Chilean IPNV isolates, the positions showed were proposed as linked to virulence [13, 14]. ND: not determined

Amino acid position					Amino acid position				
Isolate	217	221	247	500	Isolate	217	221	247	500
Genogroup 5					Genogroup 5				
1096-BC2	T	A	T	Y	1096-V2	T	A	T	ND
1096-BC4	T	A	T	Y	1096-V3	T	A	T	ND
1096-BC5	S	S	T	ND	1096-V4	T	A	T	Y
1096-BC6	T	A	T	Y	1096-V5	P	S	A	ND
1096-BC7	T	A	T	ND	1096-V6	T	A	T	ND
1096-BC10	T	A	T	Y	1096-V8	T	A	T	Y
1096-BC11	T	A	T	ND	1096-V10	T	T	T	ND
1096-BC12	T	A	T	Y					
1096-BC13	T	A	T	ND	Genogroup 1				
1096-BC14	T	T	T	ND	1096-V1	T	A	T	ND
1096-BC16	T	A	T	H	1096-V7	A	T	A	ND
1096-BC17	T	A	T	ND	1096-V9	A	T	A	ND
1096-BC18	T	T	T	ND	1096-BC1	A	T	A	R
1096-BC20	T	T	T	ND	1096-BC3	A	T	A	ND
1096-BC21	T	T	T	ND	1096-BC8	A	T	A	ND
1096-BC22	T	T	T	ND	1096-BC9	A	T	A	ND
1096-BC23	T	T	T	ND	1096-BC15	A	T	A	ND
1096-BC24	T	T	T	ND	1096-BC26	A	T	A	ND
1096-BC25	T	S	T	ND	1096-BC27	A	T	A	ND

**Fig. 4:**
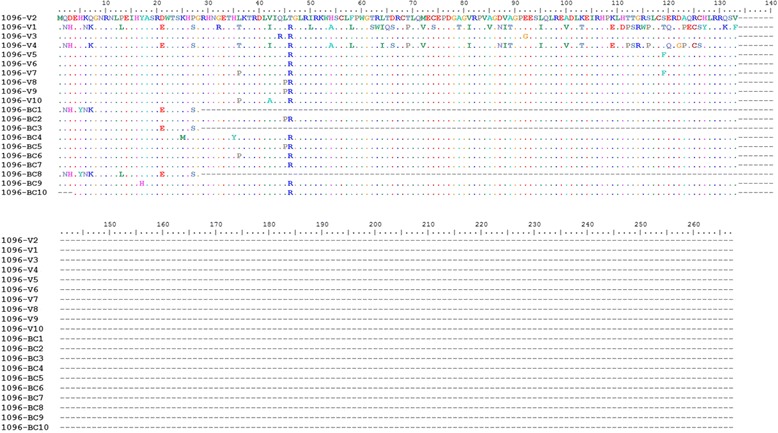
Multiple alignment of deduced amino acid sequences of VP5 belongings to isolates 1096-V1 to V10 and 1096-BC1 to BC10. Isolates of genogroup 1 and 5 were aligned for deduced amino acid sequence of VP5 by using Clustal W (Bioedit)

**Fig. 5:**
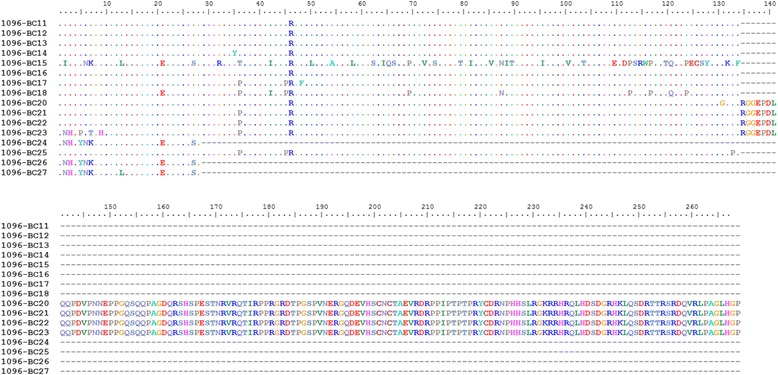
Multiple alignment of deduced amino acid sequences of VP5 belongings to isolates 1096-BC11 to BC27. Isolates of genogroup 1 and 5 were aligned for deduced amino acid sequence of VP5 by using Clustal W (Bioedit)

Comparative analyses of the VP5 deduced amino acid sequences showed that six had a truncated VP5 with 28 amino acid residues (around 3.1 kDa), five of which belonged to genogroup 1 (1096-BC1, BC3, BC8, BC26, and BC27) and one to genogroup 5 (BC24; Figs. 4 and 5). Moreover, 26 isolates showed a VP5 with 133 amino acid residues (around 14.6 kDa). Surprisingly, four isolates (1096-BC20, BC21, BC22, and BC23) had a VP5 isoform longer than 269 amino acid residues (around 29.6 kDa) (Fig. 5).

**Fig. 6:**
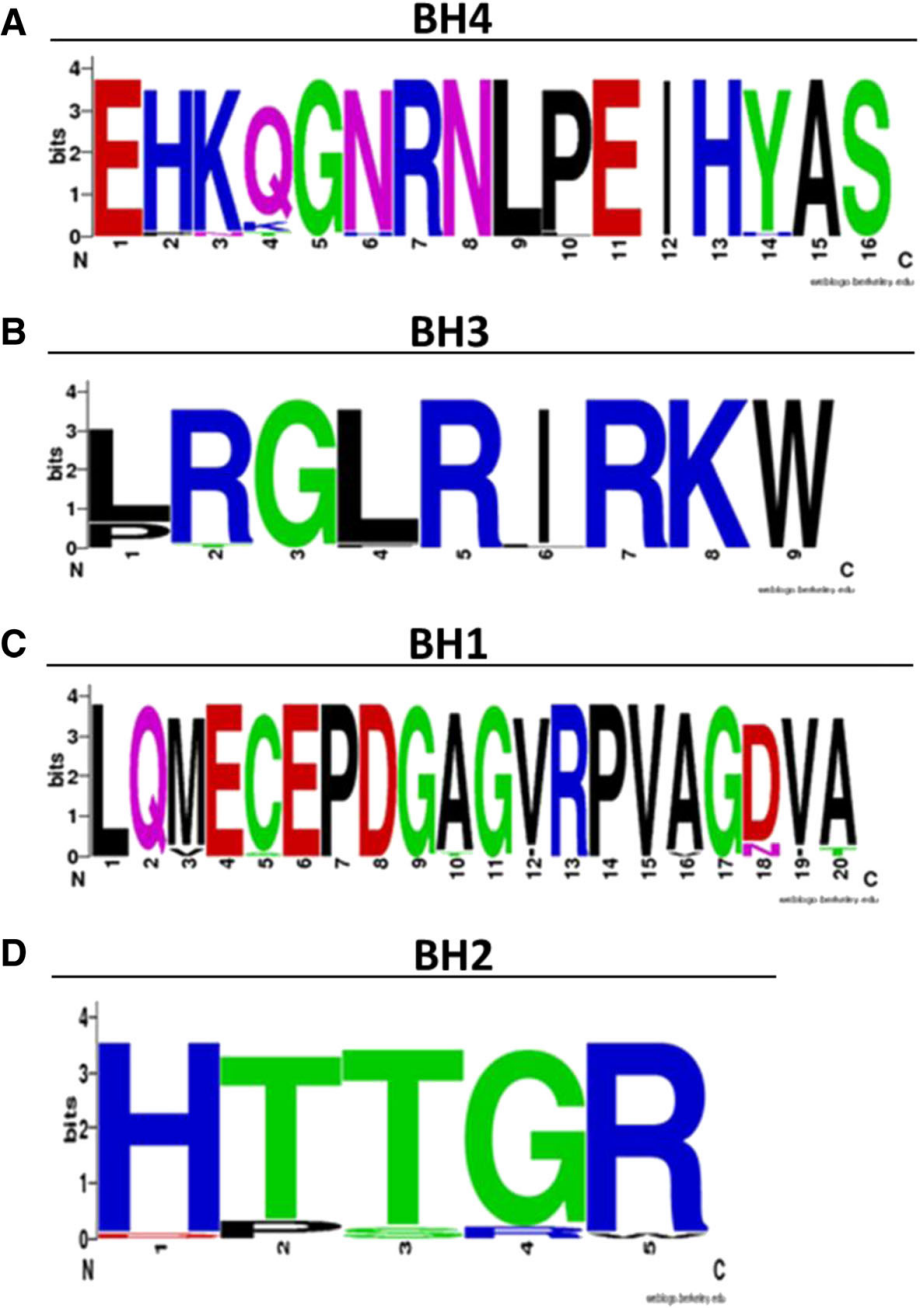
Sequence Logo diagram showing amino acid sequences of Bcl-2 Homology (BH) domains in VP5 of Chilean IPNV isolates. In A, B, C and D, consensus sequences of the BH4, BH3, BH1 and BH2 domains of VP5 in Chilean IPNV isolates, respectively. Numbers shown below the sequence represent the amino acid position in the domains. The alignment was performed using MUSCLE software. The Logo was built using the WebLogo software (http://weblogo.berkeley.edu/logo.cgi)

WebLogo analysis of the BH domains of VP5 showed that the BH4 domain had a highly conserved sequence, with slight variations present only in position 4 of this sequence (Fig. 6). Specifically, Gln was in this position in 86% of cases (Fig. 6a; larger letters in position 4), whereas Lys was in the same position in 14% of cases (Fig. 6a; the small letter K that appears below the Q in position 4). When comparing this sequence with BH4 domains from viruses and other Bcl-2-related proteins (Table 6), a very high identity to IPNV E1-S and even higher identity to MABV Y-6 were observed (Fig. 6b). Isolate analysis for BH3 was similar to that described above for BH4, with a high identity value to the BH3 domain of MABV Y-6. In slight contrast, the sequence Logo for the BH1 domain in Chilean IPNV isolates (Fig. 6c) was more similar to IPNV E1-S than MABV Y-6. Finally, the situation for the BH2 domain was very different (Fig. 6d). The principal sequence Logo obtained for the Chilean VP5 sequences did not present similarity with any of the BH2 domains used as canonical BH domain sequences (Table 6).

**Fig. 7:**
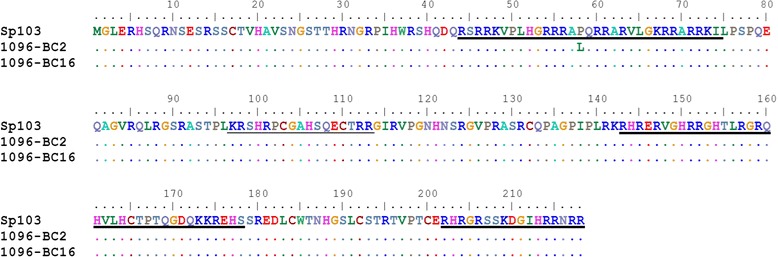
Alignment of deduced amino acid sequence of putative 25 kDa protein of Sp103 strain of IPNV and Chilean IPNV isolates BC2 and BC16. Potential nuclear targeting sequences in IPNV are underlined. The predicted nuclear localization sequences from this study are underlined with a thick line. One of NLS described by Shivappa et al. [12] was also included and is underlined with a thin line

**Table 6:**
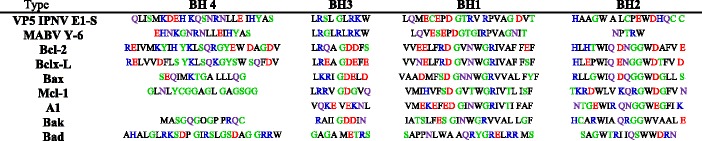
Highly conserved Bcl-2 homology (BH) domain 1–4. The table shows amino acids from the conserved BH4, BH3, BH1, and BH2 domains of VP5 for IPNV E1-S (Ab strain) and marine birnavirus Y-6 (MABV Y-6), and Bcl-2 family members

For the nine isolates in which segment A was fully sequenced, analysis showed that only two (1096-BC2 and BC16) evidenced the existence of an ORF at position 1326 to 1982 from the VP2 start, coding for a complete additional 25 kDa protein, as previously described by Shivappa et al. [12]. The remaining isolates presented a truncated sequence at position 38 of the putative 25 kDa protein (data not shown). According to alignment analyses, and using the online NLS_Mapper and NLStradamus tools, four possible NLS sequences were recognized in the putative 25 kDa protein of the Chilean isolates (Fig. 7), which were also present in the nonstructural proteins of the *Drosophila* X virus and IPNV strain Sp103 [12].

## Discussion

In 2015, Chile produced 834,000 t of salmonids. This production was the result of 726 freshwater/seawater culturing centers distributed along a distance covering roughly 2000 Km. Due to the economic importance of this industry, and considering the underreported presence of IPNV, a national characterization of the different IPNV strains present in Chile is of considerable importance. In this study, 36 Chilean IPNV isolates were characterized following sample collection over a 5-year period. Samples were collected from Atlantic salmon, rainbow trout, and coho salmon, the three most important salmonid exports for Chile.

In accordance with previous reports, only two types of Chilean-isolated IPNV strains were found. Specifically, the obtained isolates were classified as either genogroup 1, which is highly similar to the American reference strain West Buxton, or genogroup 5, which is highly similar to the European reference strain Sp. Most of the genogroup 1 isolates originated from the Los Lagos Region in southern Chile, but some were also of isolated from sites in central and the extreme-south of Chile. Genogroup 5 isolates were present in all of the analyzed regions (Table 1).

Amino acid sequence analysis showed two independent groups with 100% identities within genogroup 5: BC20 (*S. salar*), BC21 (*O. kisutch*), and BC23 (*O. kisutch*); and BC11 (*S. salar*) and BC13 (*S. salar*). Only BC11 and BC13 maintained 100% nucleotide identity, whereas BC20, BC21, and BC23showed identities ranging from 99.24 to 99.58%. Interestingly, the 1096-BC11 and BC13 isolates were obtained from different geographical zones: the Aysén (72.7° S; 45.4° W) and Los Lagos (72.3° S; 41.5° W) Regions, respectively. Nevertheless, since data on egg sources were unavailable, a single egg origin for both locations cannot be ruled out.

Correlation analysis between the geographic location and phylogenetic characteristics of the outbreaks can provide important information to understand viral patterns of spreading or mobility throughout salmonid farms [42]. For the isolates 1096-BC20, 1096-BC21, 1096-BC22, 1096-BC23, and 1096-V10, which constituted a clade of genogroup 5, at least three (1096-BC20, 1096-BC22, and 1096-BC23) had a common geographical origin (Aysén Region; Fig. 2c). These three isolates were obtained in 2009, as was isolate 1096-BC21, which originated from roughly 500 miles (800 Km) away in the Araucania Region (Fig. 2a). The 1096-BC21 isolate came from an *O. kisutch* hatchery, but as there is no public record for salmonid transport between farms, it is unknown if fry from this hatchery were later transported to the Aysén Region for the next phase of growth. The 1096-V10 isolate was detected in the Los Lagos Region, a midway point between the Araucania and Aysén Regions (Fig. 2b). Nevertheless, 1096-V10 was obtained from a *S. salar* fry at a hatchery in 2006. Curiously, 1096-BC20, 1096-BC22, and 1096-BC23 were isolated from juvenile/adult *O. kisutch* and *S. salar*. The other clade within genogroup 5 was composed by 1096-BC2, 1096-BC5, and 1096-BC14. Of these, 1096-BC2 and 1096-BC14 were isolated in 2010 from the Comau Fjord (Los Lagos Region), while 1096-BC5 was isolated in 2009 from the Aysén Region. Once again, uncertainty remains regarding the transport of *S. salar* fry and juveniles between the Aysén Region and Comau Fjord. Regarding the genogroup 5 clade formed by 1096-BC11, 1096- BC13, and 1096-BC16, all isolated in 2010, two came from Cisnes (Aysén Region), while 1096-BC13 came from the Reloncaví Estuary. While there is no certainty that the fish came from the same hatchery, 1096-BC11 and 1096-BC13 did have identical nucleotide sequences. Other clades within genogroup 5 were even more difficult to correlate. Aquaculture activities in Chile have existed for more than three decades, and the massive transport of salmonids has always occurred between regions. This fact could explain, for example, why the 1096-V6 virus, isolated in 2006, was close to the 1096-V5 virus isolated in 2008, since both were from the Los Lagos Region. Similarly, it might clarify how the 1096-V4 virus, isolated in 2008 in the Los Ríos Region, could be closely related to the 1096-BC24 virus isolated in 2009 in the Aysén Region.

On the other hand, the genogroup 1 isolates were represented in most regions, except the Los Ríos and Magallanes Regions, although this could be due to the overall low number of isolates obtained from these areas. Both clades observed in this genogroup showed a clear relatedness to the isolation year rather than to region: Isolates 1096-V1, 1096-V7, and 1096-V9, which constituted one clade, were isolated in 2006, while the clade composed by isolates 1096-BC1, 1096-BC3, 1096-BC8, 1096-BC9, 1096-BC15, and 1096-BC26 was sampled from 2009 to 2010 in the Maule and Aysén Regions, which are separated by more than 700 miles (1130 Km).

In contrast to Tapia et al [30], the present study found genogroup 1 isolates (American origin) not only in *O. mykiss*, but also in *O. kisutch* and *S. salar*. Similarly, genogroup 5 isolates (European origin) were present not only in *S. salar*, but also in the other two salmonid species. These findings suggest that in Chile there is no fixed relationship between salmonid species and genogroups. Nevertheless, most isolates from *S. salar* (over 60%) belonged to genogrup 5, while *O. mykiss* and *O. kisutch* were equally represented (around 20% each) in that genogroup.

Unlike isolates from genogroup 5, the 10 isolates from genogroup 1 were obtained only in samples from freshwater and estuary fish. However, considering the low number of cases for these isolates, it would be unwise to assume that Chilean genogroup 1 IPNV isolates do not infect seawater salmonids.

In relation to VP2 amino acid residues related to virulence described for Sp strains, the motifs Thr217, Ala221, and, to a lesser extent, Thr247 and His/Tyr500 are associated with high virulence, whereas the motifs Pro217, Ala221, and Ala247 are related to low/moderate virulence [12–14]. In the current study, more than half of the isolates in genogroup 5 presented high virulence motifs (i.e. Thr, Ala, and Thr at positions 217, 221, and 247, respectively), and seven showed Tyr at position 500. These isolates contrasted with eight genogroup 5 isolates that presented Thr at position 221, which is associated with almost avirulent strains and the establishment of a carrier state or persistent IPNV infection in salmonids [13, 14, 18]. Unfortunately, data on mortality percentages in field cases are frequently unavailable, either because the information provided by the farmer is imprecise or because there is a policy to not provide such information. Although the laboratory managers that provided the isolates used in the present study claimed that the isolates came from virulent outbreaks, precise information was obtained only for two: 1096-V2 and 1096-V4, with high (about 80%) and medium (40%) level of mortality in field, respectively. Interestingly, these two Sp isolates presented the motifs associated with high virulence (i.e. Thr217, Ala221, Thr247), and 1096-V4 also presented Tyr at position 500.

On the other hand, possible virulence markers for VP2 amino acids were not found associated with genogroup 1 strains. Nine of ten genogroup 1 isolates classified in the current study presented Ala, Thr, and Ala at positions 217, 221, and 247, respectively. Although these isolates supposedly came from virulent outbreaks that were duly reported for diagnosis and identification, accurate information about resulting mortality percentages was unavailable. This amino acid arrangement has been frequently found, either partially (A217 and T221) or completely, in most previously described genogroup 1 isolates [30, 43]. Interestingly, two isolates (1096-V5 [*S. salar*] and BC25 [*S. salar*]) presented Ser residues at position 221, while the 1096-BC5 (*O. kisutch*) isolate presented this amino acid at positions 217 and 221. The presence of serine at position 221 was previously described for one Chilean IPNV isolate [30]. Moreover, only one isolate presented Pro at the position 217, which is in contrast to that normally described for moderate- to low-virulence Sp isolates obtained from fish or eggs originating from Ireland, Scotland, Norway, or Spain [12, 29, 44–46].

Recently, Mutoloki et al. [47] described two different VP2 amino acid motifs, or fingerprints, for IPNV associated with clinical and subclinical infections: I64T137T217A221T247V252T281N282A319 and V64A137P217T221A247N252S281D282E319, respectively. A search for these fingerprints in the VP2 amino acid sequences of the isolates evaluated in this study showed that of the 26 isolates belonging to genogroup 5, 13 presented the complete motif associated with the clinical fingerprint. Of the remaining 13, seven presented T221 as the only substitution with respect to the clinical fingerprint, while a single isolate presented A247 as the only substitution. Three isolates had two substitutions, and two isolates had three substitutions. In most cases, the substitutions corresponded to amino acids present in the motif associated with the subclinical fingerprint, except for the two isolates presenting S221 and that of S217S221. In the case of the ten genogroup 1 isolates, all presented T221A247N252 and seven presented V64T221A247N252E319. In both cases, these corresponded to partial subclinical fingerprint sequences. Additionally, all genogroup 1 isolates presented T137 and T281, replacements associated with the clinical fingerprint. Two isolates also had I264, and one had A319, both of which are also associated with the clinical fingerprint. Furthermore, all isolates had A217 and A282, substitutions not associated with any fingerprint. Therefore, the clinical finger was only present in the genogroup 5 isolates, with very few amino acid substitutions. In turn, genogroup 1 isolates presented sequences mostly associated with the subclinical fingerprint, with practically constant amino acid substitutions in all isolates. In accordance with the above, the genogroup 5 isolates 1096-V2 and 1096-V4, with high and medium mortality levels in the field, presented amino acid motifs associated with the clinical fingerprint.

Analysis of VP5 showed that four isolates from genogroup 5 (1096-BC20 [*S. salar*], BC21 [*O. kisutch*], BC22 [S. salar], and BC23 [*O. kisutch*]) had undescribed deduced proteins with unusually long amino acid sequences. Although the relatedness among these four isolates was demonstrated by phylogenetic analysis, they were isolated from different regions, fish species, and environments. Therefore, establishing any relationship is unlikely. The role of these longer isoforms in VP5 is unknown. All VP5 amino acid sequences were analyzed using the online tools TMpred and TOPCONS, which detect characteristic topologies in membrane proteins or peptides and determine the presence of transmembrane domains. Results showed an absence of transmembrane sequences, in accordance with previous classification as an anti-apoptotic protein class III [48]. Moreover, to determine the presence of motifs for plasma membrane tropism, the VP5 sequences of the obtained Chilean isolates and of the infectious bursal disease virus were searched for the presence of polycationic regions [36], the signals of which were not found in the Chilean isolates.

The WebLogo analysis established that the deduced amino acid sequences of the VP5 BH4, BH3, and BH1 domains of the Chilean IPNV isolates were highly similar to corresponding BH domains in MABV Y-6 and, to a slightly lesser extent, IPNV E1-S (Ab strain) [39]. However, the principal Logo sequence obtained for BH2 was not similar to any of the selected canonical sequences. Nonetheless, combining the Logo sequences of HTTGR (with higher percentage of conservation, Fig. 6d, big letter) and DPSRW (with lowest percentages of conservation, Fig. 6d, letters below the largest) obtained a partial match with the NPTRW sequence, the BH2 domain described for MABV Y-6 (Table 6). Nevertheless, this pattern was only present in two genogroup 1 isolates and partially in one genogroup 5 isolates, which is very unrepresentative. Clearly, the Logo sequences of the Chilean isolates were unrelated to the canonical BH domain sequences present in class I (Bcl-2, Bcl-xL, and Mcl-1) and class II (Bcl-w and A-1) anti-apoptotic proteins, nor with the BH sequences of pro-apoptotic proteins (Bax, Bak and Bad) [39, 48]. Namely, VP5 sequences of Chilean IPNV isolates were highly similar to the marine birnaviruses MABV Y-6 and IPNV E1-S, an Ab serotype, in addition to being unrelated with anti- or pro-apoptotic proteins.

Shivappa et al. [12] described the presence of an ORF for a putative 25 kDa protein located between the VP2 and VP4 coding regions. When assessing the sequences coding for this putative protein in the Chilean viral isolates, only two of the nine isolates with a fully sequenced segment A coded for the complete sequence of the putative 25 kDa protein. According to the performed nuclear localization signal (NLS) alignment analysis against the putative 25 kDa protein of the IPNV strain Sp103, previously investigated by Shivappa et al. [12], two of the described sequences coincided with the two assessed Chilean isolates, with the first between amino acid residues 46 to 71 and the second predicted between residues 97 to 113. Moreover, prediction analyses using the online tools NLS_Mapper and NLStradamus revealed that only the first would correspond to a NLS. These tools also detected two additional putative NLS in both Chilean isolates between residues 143 to 178 and 202 to 218 (Fig. 7). This finding partly coincided with previous descriptions in the nonstructural protein of the *Drosophila* X virus and the IPNV strain Sp103 [12]. The presently identified NLS sequences were characterized by the presence of polycationic regions that spanned from the middle of the protein towards the carboxyl terminal. A wide variety of cellular proteins employ surface-exposed, positively charged domains to interact with anionic lipids anchored to the inner plasmatic membrane leaflet [49, 50]. Moreover, the TargetP 1.1 predictor was used to supplement information regarding other possible subcellular location signals in the putative 25 kDa protein. This analysis predicted the presence of a 61 amino acid N-terminal presequence for mitochondrial targeting in the isolates 1096-BC2 and 1096-BC16 (data not shown). The presence of such signals has been previously described for several viral proteins. This situation appears to be similar to what happens with the non-structural Borna disease virus protein X, which is targeted to both the nucleus and mitochondria of infected cells and inhibits the aggregation of the mitochondrial antiviral-signaling protein, thus blocking programmed cell death and confering resistance to Fas-induced cell death [51]. Clearly, more investigative progress is needed for the putative protein of 25 kDa, mainly regarding detection as a protein in infected cells, but also on its role in relation to potential nuclear and mitochondrial targeting.

## Conclusions

This study molecularly characterized 36 Chilean IPNV isolates sampled over 6 years from the three most cultured salmonids in Chile. The obtained results represent an important advancement in the analysis of IPNV strains present in Chile, providing information about phylogenetic relationships, geographical areas, tropism for salmon species, and characteristics of the VP2, VP5, and putative 25 kDa proteins. Moreover, the obtained data are of upmost relevance for productive salmonid management, providing information that might help in identifying epidemiological links and in developing specific sanitary tools for preventing IPNV risks in Chilean aquaculture.
